# Lack of correlation between pulmonary disease and cystic fibrosis transmembrane conductance regulator dysfunction in cystic fibrosis: a case report

**DOI:** 10.1186/1752-1947-4-117

**Published:** 2010-04-26

**Authors:** Hara Levy, Carolynn L Cannon, Daniel Asher, Christopher García, Robert H Cleveland, Gerald B Pier, Michael R Knowles, Andrew A Colin

**Affiliations:** 1 Division of Pulmonary Medicine, Children's Hospital Boston, 300 Longwood Avenue, Boston, USA; 2 Harvard Medical School, 25 Shattuck Street, Boston, USA; 3 Channing Laboratory, Brigham and Women's Hospital, 181 Longwood Avenue, Boston, USA; 4 Division of Allergy and Pulmonary Medicine, St. Louis Children's Hospital, One Children's Place, St. Louis, USA; 5 Division of Radiology, Children's Hospital, 300 Longwood Avenue, Boston, USA; 6 University of North Carolina School of Medicine, Division of Pulmonary and Critical Care Medicine,209 Boulder Bluff Trail Wolfs Pond, Chapel Hill, USA; 7 Division of Pediatric Pulmonary Medicine, Miller School of Medicine, University of Miami, 1580 Northwest 10th Avenue, Miami, USA

## Abstract

**Introduction:**

Mutations in both alleles of the cystic fibrosis transmembrane conductance regulator gene result in the disease cystic fibrosis, which usually manifests as chronic sinopulmonary disease, pancreatic insufficiency, elevated sodium chloride loss in sweat, infertility among men due to agenesis of the vas deferens and other symptoms including liver disease.

**Case presentation:**

We describe a pair of African-American brothers, aged 21 and 27, with cystic fibrosis. They were homozygous for a rare frameshift mutation in the cystic fibrosis transmembrane conductance regulator 3791delC, which would be expected to cause significant morbidity. Although 80% of cystic fibrosis patients are colonized with *Pseudomonas aeruginosa *by eight years of age, the older brother had no serum opsonic antibody titer to *P. aeruginosa *by age 13 and therefore would have failed to mount an effective antibody response to the alginate (mucoid polysaccharide) capsule of *P. aeruginosa*. He was not colonized with *P. aeruginosa *until 24 years of age. Similarly, the younger brother was not colonized with *P. aeruginosa *until age 20 and had no significant lung disease.

**Conclusion:**

Despite a prevailing idea in cystic fibrosis research that the amount of functional cystic fibrosis transmembrane conductance regulator predicts clinical status, our results indicated that respiratory disease severity in cystic fibrosis exhibits phenotypic heterogeneity. If this heterogeneity is, in part, genetic, it is most likely derived from genes outside the cystic fibrosis transmembrane conductance regulator locus.

## Introduction

Mutations in both alleles of the cystic fibrosis transmembrane conductance regulator (CFTR) gene result in the disease cystic fibrosis (CF), which manifests classically as chronic sinopulmonary disease, pancreatic insufficiency, elevated sodium chloride loss in sweat, infertility among men is due to agenesis of the vas deferens and other symptoms like liver disease. Except for patients with significant liver disease, the primary disease morbidity is linked to the chronic pulmonary infections and consequent decline in lung function. CFTR mutations are classified as severe (class I-III mutations) or mild (class IV-V mutations) based on their effect on protein synthesis and function, implying that the less CFTR that is made or is functional, the more severe the clinical course of a patient with cystic fibrosis (CF) [1-4]. Importantly, none of the CFTR mutations correlate with sweat chloride levels and only few of the more than 1,500 identified mutations in CFTR result in an expected respiratory disease phenotype in homozygous or compound heterozygous patients. It is well-accepted that the diversity of lung disease among CF patients is not accounted for either by variation in CFTR mutations or by level of sweat chloride as there is considerable phenotypic heterogeneity even in patients with the same class of CFTR mutation. In this report, we describe a pair of siblings with a mild CF phenotype, who are homozygous for the 3791delC mutation, a rare CFTR frameshift mutation found originally in an African American patient with CF [5,6]. The review was approved by the Children's Hospital's Institutional Review Board. On the basis of its classification as a severe mutation, the 3791delC mutation is expected to cause significant morbidity, yet these brothers present with an incongruously mild clinical course.

## Case presentation

We present two African American brothers aged 24 and 20 years. Both siblings were diagnosed with CF based on symptoms and confirmed by sweat iontophoresis. The elder brother presented with meconium ileus at birth. His sweat chloride level was 104 meq/L. His brother, who is six years younger, had a sweat chloride level of 113 meq/L. Both are pancreatic insufficient, as documented by low stool elastase levels, but, with appropriate nutritional, vitamin and enzyme supplementation. Each maintains a BMI (body mass index) of 24.0. Genotyping (Genzyme Corporation, Cambridge, MA and Ambry Genetics, Aliso Viejo, CA) verifies homozygosity for the 3791delC mutation. The semen analysis from older brother showed no sperm. The younger brother, meanwhile, had no semen analysis. Neither parent was available to give family history concerning consanguinity or blood samples for genotyping.

**Figure 1:**
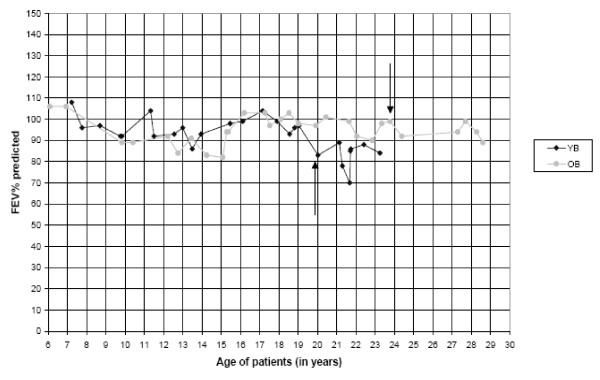
**Pulmonary Function (PFT) FEV_1_% predicted**. Each line depicts the PFT values for each sibling over the course of 14 years. Arrows depict first culture documentation of *Pseudomonas aeruginosa*. OB = older sibling; YB = younger sibling.

While 80% of CF patients are colonized with *Pseudomonas aeruginosa *by eight years of age [7,8], the older brother, by age 13, had no serum opsonic antibody titer to *P. aeruginosa *and therefore would fail to mount an effective antibody response to the alginate (mucoid polysaccharide) capsule of *P. aeruginosa*. Still, he was not colonized with *P. aeruginosa *until he reached 24 years of age. Similarly, the younger brother was not colonized with *P. aeruginosa *until he was 20 years old and had no significant lung disease. Figure 1 shows pulmonary function results over 14 years and decline in pulmonary function since colonization with *P. aeruginosa*. (See additional file 1: Figure 1: PFT_FEV1%_Predicted for pulmonary function results.) Except during two endobronchial exacerbations each, FEV_1 _is >80% predicted, consistent with the top quartile and normal lung function for age according to the Epidemiological Study of Cystic Fibrosis (ESCF) classification [9]. The brothers had sequential chest X-rays (CXRs) scored by the Brasfield system [10] with sequential FEV_1 _and FVC, by a predictive scoring system developed from a large CF cohort [11,12] One brother had 14 CXRs over a 12.5-year period (age four months to 12 years and nine months); the other had 20 CXRs over 18 years (age one month to 18 years, three months). The brothers' aggregate decline in CXR score was 0.027%/year, FEV_1 _-0.018%/year, and FVC -0.012/year, compared with aggregate declines for 57 patients homozygous for the ΔF508 CFTR mutation in the same cohort of CXR scores -0.065%/year, FEV_1 _-0.045%/year and FVC -0.044%/year (unpublished data). While the brothers presented with the classical symptoms of CF and are homozygous for a CFTR mutation that should predict a severe CF phenotype, they have a mild clinical course.

## Discussion

According to CFTR nomenclature and sequence data, 3791delC is a frameshift CFTR mutation causing deletion of the second base, cytosine, of codon 1220 in the CFTR gene. This results in substitution of amino acids 1220 through 1226 in wild-type CFTR, TEGGNAI for KKVEMPY, followed by production of a stop codon, UAG, at position 1227, resulting in a truncated protein.

One case report [13] describes two CF patients with a nonsense mutation in CFTR and mild pulmonary disease. But there are no reports of *in vitro *functional analysis of this 3791delC CFTR mutation. Thus, the conclusions we draw regarding function of the 3791delC mutant CFTR are by analogy to truncation mutants. Residue 1219 is the first amino acid of the second nucleotide-binding domain (NBD2) of CFTR. Numerous investigators have examined the functionality of NBD2 mutants and C-terminal CFTR truncation mutants. Although some truncation mutants have normal maturation, most exhibit accelerated degradation of mRNA and protein and aberrant trafficking similar to ΔF508 CFTR [14] resulting in a significant reduction in chloride channel activity [15]. Portions of CFTR may dimerize and make some functional CFTR within the respiratory epithelium. However, most mutations that lead to premature termination signal cause nonsense-mediated mRNA decay and, consequently, absence of protein synthesis. These properties predict a severe phenotype, especially in homozygous mutants. However, our patients have a mild CF phenotype.

## Conclusion

A prevailing idea in CF research is that the amount of functional CFTR predicts clinical status; research focus on gene therapy and upregulation of CFTR is based on this premise. Our results indicate that disease severity in CF can be variable even in patients with a CFTR mutation that produces absent or aberrant protein. Ultimately, we found a lack of correlation between the CFTR mutation classification and lung function, which is likely partially due to differential CFTR activity between the sweat gland and lung epithelium, as well as the activity of modifier genes and proteins. We suspect that the function of the CFTR membrane transporter is entirely different (that is, a non-transport function) or may differ with respect to a substrate. Applicably, besides the transport of substrates such as chloride and glutathione, CFTR has non-transport functions, as illustrated by its role as a receptor for *P. aeruginosa*. Thus, differing effects of each mutation on CFTR function may account for some of the phenotypic heterogeneity and lack of correlation between CFTR mutation and clinical course. The incongruously benign course that these siblings present despite the expectation that their 3791delC mutation produces little or no functional CFTR, implies that factors outside CFTR, likely modifier genes, have a potent compensatory effect, and can steer the course away from its predicted severity.

## Consent

Both patients were lost to follow-up and efforts to trace them and their family have proved futile. The Institutional Review Board at Children's Hospital of Boston has approved this case report for publication. Every effort has been made to keep the patients' identities anonymous and we would not expect a reasonable patient or their family to object to publication of this case report and any accompanying images.

## Competing interests

The authors declare that they have no competing interests.

## Authors' contributions

HL analyzed and interpreted our patient data and wrote the manuscript. CLC, KCG, GBP, MRK and AAC performed critical editorial review. RHC provided the radiologic information. KCG and DA provided assistance with figure 1. All authors read and approved the final manuscript.
